# Acute Effects of Ambient PM_2.5_ on All-Cause and Cause-Specific Emergency Ambulance Dispatches in Japan

**DOI:** 10.3390/ijerph15020307

**Published:** 2018-02-09

**Authors:** Vera Ling Hui Phung, Kayo Ueda, Shunji Kasaoka, Xerxes Seposo, Saira Tasmin, Shinichi Yonemochi, Arthit Phosri, Akiko Honda, Hirohisa Takano, Takehiro Michikawa, Hiroshi Nitta

**Affiliations:** 1 Department of Environmental Engineering, Graduate School of Engineering, Kyoto University, Kyoto 615-8540, Japan; phung.hui.62a@st.kyoto-u.ac.jp (V.L.H.P.); seposo@health.env.kyoto-u.ac.jp (X.S.); rimjhim1612@gmail.com (S.T.); phosri.arthit.82v@st.kyoto-u.ac.jp (A.P.); akko@health.env.kyoto-u.ac.jp (A.H.); htakano@health.env.kyoto-u.ac.jp (H.T.); 2 Emergency and General Medicine, Kumamoto University Hospital, Kumamoto 860-8556, Japan; kasaoka@fc.kuh.kumamoto-u.ac.jp; 3 Center for Environmental Science in Saitama, Kazo, Saitama 347-0115, Japan; yonemochi.shinichi@pref.saitama.lg.jp; 4 Center for Health and Environmental Risk Research, National Institute for Environmental Studies (NIES), Tsukuba, Ibaraki 305-8506, Japan; tmichikawa@nies.go.jp (T.M.); nitta@nies.go.jp (H.N.)

**Keywords:** air pollution, ambient PM_2.5_, emergency ambulance dispatches, short-term exposure

## Abstract

Short-term health effects of ambient PM_2.5_ have been established with numerous studies, but evidence in Asian countries is limited. This study aimed to investigate the short-term effects of PM_2.5_ on acute health outcomes, particularly all-cause, cardiovascular, respiratory, cerebrovascular and neuropsychological outcomes. We utilized daily emergency ambulance dispatches (EAD) data from eight Japanese cities (2007–2011). Statistical analyses included two stages: (1) City-level generalized linear model with Poisson distribution; (2) Random-effects meta-analysis in pooling city-specific effect estimates. Lag patterns were explored using (1) unconstrained-distributed lags (lag 0 to lag 7) and (2) average lags (lag: 0–1, 0–3, 0–5, 0–7). In all-cause EAD, significant increases were observed in both shorter lag (lag 0: 1.24% (95% CI: 0.92, 1.56)) and average lag 0–1 (0.64% (95% CI: 0.23, 1.06)). Increases of 1.88% and 1.48% in respiratory and neuropsychological EAD outcomes, respectively, were observed at lag 0 per 10 µg/m^3^ increase in PM_2.5_. While respiratory outcomes demonstrated significant average effects, no significant effect was observed for cardiovascular outcomes. Meanwhile, an inverse association was observed in cerebrovascular outcomes. In this study, we observed that effects of PM_2.5_ on all-cause, respiratory and neuropsychological EAD were acute, with average effects not exceeding 3 days prior to EAD onset.

## 1. Introduction

Compared to studies of mortality, there are fewer studies examining the association between short-term exposure to ambient fine particulate matter (PM_2.5_) and morbidity, such as hospitalization and emergency visits [1,2,3,4,5,6]. Multi-city studies, particularly, are scarce in the Asian region [7]. More studies in Asia, especially multi-city studies, would contribute to the comparability of air pollution-related health effects with previous studies conducted in western countries [8].

Hospitalization and emergency visits are commonly-used health indicators in reporting the association of acute morbidity and ambient air pollution. However, the database which records daily morbidity information over multiple areas for epidemiological studies has not been fully established in some countries. The use of emergency ambulance dispatches (EAD) as a morbidity indicator in studies investigating health effects of ambient air pollutants has been increasing in recent years [9,10,11,12,13,14]. This has the potential to act as a proxy for health outcomes, especially in countries without detailed health databases. Only a few studies have examined PM_2.5_ health effects using EAD data [10,12,13,14]; the others examined the health effects with PM_10_ [9] and suspended particulate matter (SPM) [11]. In addition, most of the studies estimated the association in single-city settings [10,13,14]. Although currently published studies using EAD data, including a study from Japan with a limited study period [12], have suggested that there are associations between PM_2.5_ and all-cause, respiratory, and cardiovascular outcomes [13,14], there is limited evidence examining the association between PM_2.5_ and EAD due to various health outcomes in a multi-city setting.

Recent studies have reported on the effects of PM_2.5_ on cardiopulmonary outcomes [7], with the possible causal pathway linked to the underlying mechanism of systemic inflammatory responses and oxidative stress [15]. Previous studies carried out in Japan [10,12], however, reported weak evidence of PM_2.5_ effects on cardiovascular outcomes. In addition, it was previously observed that the effects of PM_2.5_ on neuropsychological outcomes can either be acute [4,16,17] or prolonged [18,19,20], with a majority of these effects largely underestimated [21]. Pathological pathways linked to the associated PM_2.5_ effects were suggested to be facilitated through inflammatory responses, oxidative stress, microglial activation, cerebrovascular dysfunction, and alterations in the blood–brain barrier (BBB) [22].

In light of these findings, this study aimed to investigate the association of short-term exposure to ambient PM_2.5_ on EAD among different types of diagnoses in Japan. The included types of diagnoses were all-cause, cardiovascular, respiratory, cerebrovascular, and neuropsychological EAD outcomes.

## 2. Materials and Methods

### 2.1. Study Area

**Figure 1:**
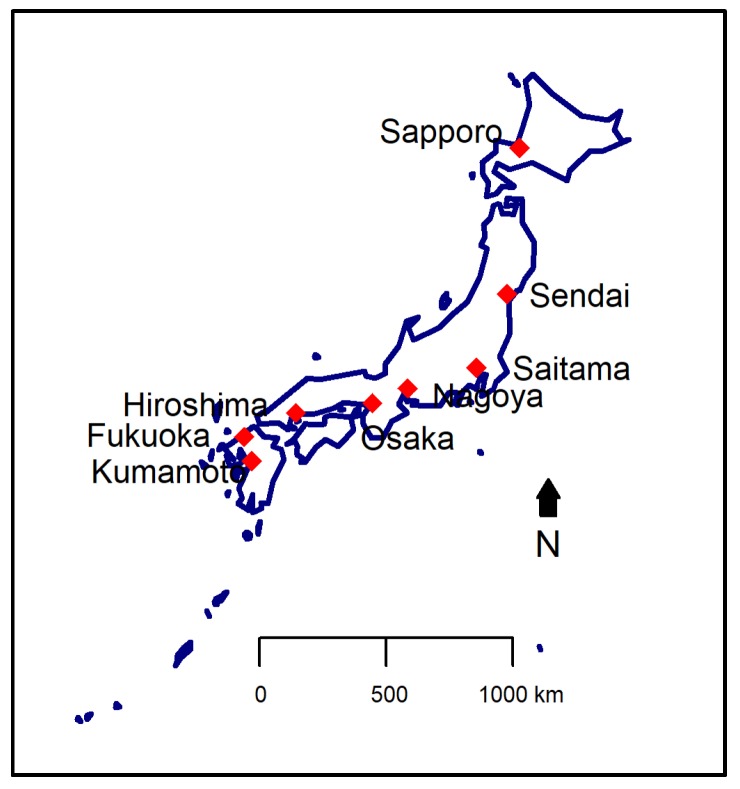
Map of Japan showing the eight cities included in this study.

This study included data from eight cities in Japan (Figure 1), spanning from 2007 to 2011. The eight cities are located distinctly across the northern to the southern regions. The study period of each city is shown in Table 1.

### 2.2. Health Data: Daily EAD

In Japan, EAD is a free-of-charge service provided by the local fire departments, whereby the public are able to utilize the service by calling the emergency number “119” [23]. Data on daily EAD were obtained from the Fire and Disaster Management Agency of the Ministry of Internal Affairs and Communications in Japan. EAD data for Kumamoto city were later obtained from its city office. The data contained information on sex, age category, cause of dispatches, type of diagnoses, and the severity of the patient’s condition. To avoid the inclusion of health outcomes irrelevant to the main exposure, among the 14 categories for the cause of dispatches, only the category “acute illnesses” (referring to non-traumatic or non-accidental health outcome) was extracted and used for the analyses in this study. Meanwhile, primary diagnoses were determined by emergency medical doctors upon arrival at the hospital and were coded into 10 major categories (cardiovascular, respiratory, cerebrovascular, digestive, psychiatric, nerves and sensory, urological, neoplasms, others, and unknown), defined by the International Classification of Diseases, Tenth Revision (ICD-10). We further examined the association between ambient PM_2.5_ and EAD for all-cause acute illnesses (i.e., all diseases except traumatic cases), and included only EAD for cardiovascular (ICD-10: I10–I15, I20–I25, I01–I02.0, I05–I09, I27, I30–I52), respiratory (ICD-10: J00–J99), cerebrovascular (ICD-10: I63, I69.3, I60–I62, I64–I68, I69.0–I69.2, I69.4–I69.8) and neuropsychological (ICD-10: F00–F99, G00–G99, H00–H59, H60–H95) outcomes for cause-specific analyses. This study was approved by the Ethics Committee of the Kyoto University Graduate School of Engineering (No. 201410).

### 2.3. Environmental Data

Hourly concentrations of ambient PM_2.5_ and gaseous pollutants (nitrogen dioxide (NO_2_), photochemical oxidants (O_x_) and sulfur dioxide (SO_2_)) monitored at one background monitoring station of each city, were obtained from the National Institute for Environmental Studies (NIES). Ambient PM_2.5_ were measured using monitors that employ tapered element oscillating microbalances (TEOM) in all cities except Osaka and Saitama. Data for Osaka were based on a combination of beta ray attenuation (1 January 2008–31 March 2009) and TEOM (1 April 2009–31 December 2011). Ambient PM_2.5_ data for Saitama were obtained from the Center for Environmental Science in Saitama, based on daily filter sampling. O_x_ comprises ozone and peroxyacetyl nitrate generated by photochemical reactions (only those capable of isolating iodine from neutral potassium iodide, excluding nitrogen dioxide). Weather data (ambient temperature and relative humidity) were obtained from the Japan Meteorological Agency. Daily 24-h average concentration was calculated for each pollutant. We excluded the days which consisted of more than four missing hourly concentrations of PM_2.5_.

Data on influenza epidemics were obtained from the Japan National Institute of Infectious Diseases. A week was defined as an influenza epidemic week, when the influenza cases within that week were more than the 90th percentile of the total distribution throughout the study period [24].

### 2.4. Statistical Analyses

A two-stage analysis was conducted to estimate the relative risks of EAD associated with exposure to PM_2.5_. This included all-cause and cause-specific (cardiovascular, respiratory, cerebrovascular, and neuropsychological) EAD, and consisted of a sensitivity analysis stratifying all-cause EAD by different age groups. During the first stage, we investigated the city-specific association between ambient PM_2.5_ and daily EAD for acute illnesses, using a generalized linear model (GLM) based on a Poisson distribution. We included the following covariates, tested as per the following order: calendar date, daily mean temperature, relative humidity, public holiday, day-of-week, and influenza epidemic. The models were decided based on a model simplification approach, whereby the simpler model would be retained if the *p*-value from the *Chi-square* test of ANOVA in comparing the models was less than 0.05. A natural cubic spline was applied to calendar time to allow smooth modelling for the long-term and seasonal patterns, with 7 degrees of freedom (df) per year [25], as well as for the daily mean ambient temperature (averaged from the current day to 3 days before), with 3 df [26]. Public holidays and day-of-week were adjusted for all cities as indicator variables. Since there was no significant difference between the models with or without relative humidity, it was not included in the model for all cities. The same applied for the influenza epidemics; it was adjusted only in Sapporo and Osaka as an indicator variable.

The lagged effect of ambient PM_2.5_ was examined via an unconstrained, distributed structure, with an extension of 7 days (lag 0 to lag 7), and average structure (using average of PM_2.5_ over lag-days: lag 0–1, lag 0–3, lag 0–5 and lag 0–7) (hereafter referred to as “average lag”). The models were used to estimate the relative risk of EAD for acute illnesses per 10 µg/m^3^ increase in ambient PM_2.5_.

During the second stage, a random-effects meta-analysis was utilized to obtain the pooled effect estimates of ambient PM_2.5_ on EAD. An *I*^2^ statistic was used to estimate the amount of heterogeneity, while applying the Chi-square test from Cochran’s Q statistic to test the significance of heterogeneity [27].

To examine the robustness of the association, we applied two-pollutant models and included each gaseous pollutant in the main model one at a time. We also changed the df for calendar time (df ranged from 3 to 13) and temperature (df ranged from 4 to 8). An additional analysis was conducted with bootstrapping, utilizing 10,000 simulations to obtain the empirical estimates and confidence intervals of our results, using the estimates of all-cause EAD at lag 0–1.

All statistical analyses were conducted in R (version 3.1.1, The R Foundation for Statistical Computing, Vienna, Austria) utilizing the R packages *splines* [28] and *metafor* [29]. All results were presented as the percent change (Equation (1)) of EAD with 95% confidence interval (95% CI) per 10 µg/m^3^ increase in ambient PM_2.5_. Statistical significance was considered when the *p*-value was less than 0.05.

(1) $$\mathrm{Percent}\;\mathrm{change}=\left(exp^{estimate}-1\right)\times 100\%$$

## 3. Results

A total of 1,114,515 cases of EAD for acute illnesses in the eight cities from 2007 to 2011 were included in this study. Table 1 shows the daily average number of EAD in each city during each study period, with the lowest in Kumamoto (x̅ = 43) and highest in Osaka (x̅ = 305). Data on sex in Nagoya, Osaka, and Hiroshima were not available. Males and females were found to be almost equally distributed. The number of EAD for acute illnesses generally consisted of more elderly than children and adults. Table 2 shows the daily average value of environmental variables. The daily mean PM_2.5_ concentration ranged from 11.3 µg/m^3^ (Sapporo) to 20.8 µg/m^3^ (Hiroshima). The daily mean temperature was lowest in Sapporo (8.4 °C), while in other cities it ranged from 12.2 °C (Sendai) to 18.3 °C (Kumamoto). Tukey’s test showed that there were significant differences between cities, particularly lower PM_2.5_ concentrations and temperature in the northern cities (such as Sapporo and Sendai), compared to the other cities (Table S3).

**Figure 2:**
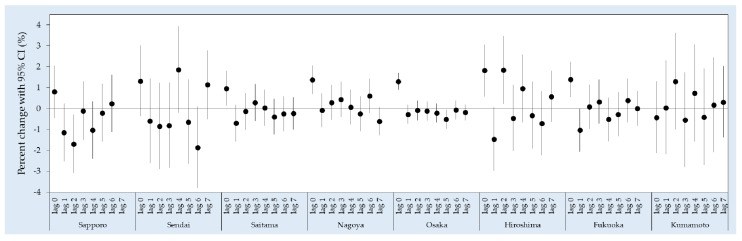
City-specific percent change of all-cause EAD associated with a 10 µg/m^3^ increase in PM_2.5_ in the unconstrained, distributed lag model.

**Figure 3:**
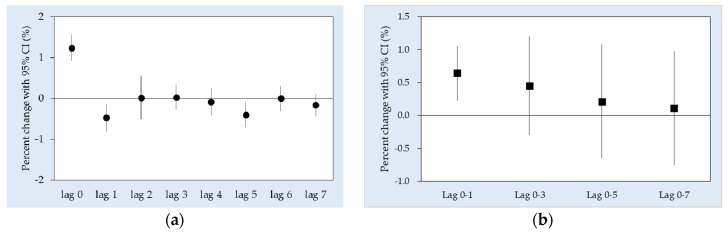
Percent change of all-cause EAD (pooled effect) associated with a 10 µg/m^3^ increase in PM_2.5_ in the (**a**) unconstrained, distributed lag, and (**b**) average lag models.

Figure 2 shows the city-specific estimates of all-cause EAD associated with PM_2.5_ from lag 0 to lag 7. In general, the highest risks of all-cause EAD were mostly observed on the current day (lag 0), especially in the cities with a larger population, i.e., Saitama, Nagoya, Osaka and Fukuoka. Pooled results for the single-day lags showed an increased risk at lag 0 (1.24% (95% CI: 0.92, 1.56)) followed by a decline at lag 1 (−0.47% (95% CI: −0.80, −0.14)) (Figure 3a). As for the average lags, an increase in EAD risk was observed at lag 0–1 (0.64% (95% CI: 0.23, 1.06)) while the effect estimates decreased at the latter lags (Figure 3b).

Cause-specific EAD outcomes due to short-term exposure to ambient PM_2.5_ are shown in Table 3. Our study showed significant increases of respiratory (1.88% (95% CI: 1.00, 2.76)) and neuropsychological (1.48% (95% CI: 0.69, 2.28)) EAD, both at lag 0. On the other hand, significant average lag effects were observed only in the respiratory outcomes (extending up to 5 days), but not in the neuropsychological outcomes. The risk of respiratory outcomes was highest at lag 0–3 (2.79% (95% CI: 1.31, 4.29)). There was no significant effect on cardiovascular outcomes, while an inverse association was observed in cerebrovascular outcomes. We observed heterogeneity at some lags but not for all categories (Table S4).

**Figure 4:**
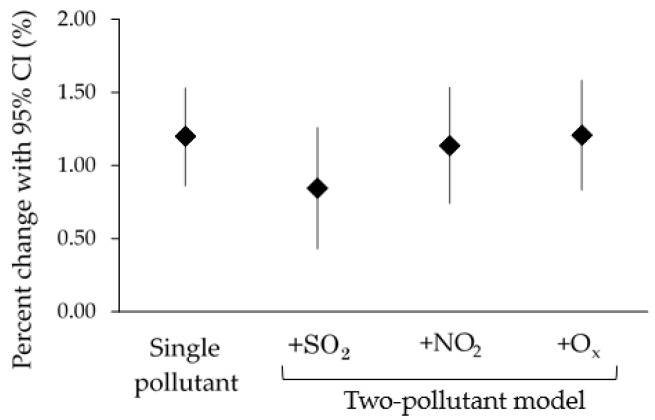
Percent change of all-cause EAD at lag 0 when adjusted using two-pollutant models. Each pollutant was included in the model one at a time.

Age-stratified results (Table 4) showed significantly increased effect estimates of all-cause EAD associated with PM_2.5_ at lag 0 in all age categories which did not differ largely among each other (children: 1.24% (95% CI: 0.21, 2.27); adults: 1.29% (95% CI: 0.87, 1.71); elderly: 1.19% (95% CI: 0.75, 1.62)). The effect estimates became smaller at the latter lags. Significantly increased effect estimates were observed in average lags for the adults (0.78% (95% CI: 0.25, 1.32) at lag 0–1) and for the elderly (0.65% (95% CI: 0.20, 1.11) at lag 0–1 and 0.61% (95% CI: 0.16, 1.07) at lag 0–3). The effect estimates of EAD for all acute illnesses remained robust in models using different df on calendar date (Table S1) and on temperature (Table S2), as well as in the two-pollutant model (Figure 4). Although there was a drop in the +SO_2_ model, the estimate remained within the 95% confidence interval of the single pollutant model, thus the drop was not significant. Nonetheless, the drop might suggest a confounding effect of SO_2_ on the association between PM_2.5_ and EAD outcomes. After bootstrapping with 10,000 simulations, the empirical estimates showed that the association between PM_2.5_ and health outcomes would always remain positive (all-cause EAD at lag 0–1: 0.49% (95% CI: 0.02, 0.98)).

## 4. Discussion

In this multi-city study, we found that short-term exposure to ambient PM_2.5_ was associated with increased risks of all-cause EAD in immediate lags (lag 0 and lag 0–1). Cause-specific analyses showed that significant associations were observed in the EAD for respiratory and neuropsychological outcomes. While the positive association persisted for longer lags and was largest at lag 0–3 for respiratory EAD, the association for neuropsychological outcomes disappeared at the latter lags.

Across cities, the PM_2.5_ levels were significantly different. PM_2.5_ levels in the northern part of Japan (Sapporo and Sendai) were generally lower than those of other cities, with the daily mean concentrations below the Japanese air quality standards for PM_2.5_ (15 µg/m^3^). Increases in EAD were observed mostly in cities with daily PM_2.5_ levels exceeding 15 µg/m^3^. However, whether the differences in PM_2.5_ levels across cities explain heterogeneity across cities requires further investigations.

It has been suggested that exposure to ambient PM_2.5_ contributes to increased risk of all-cause EAD [10,13], cardiovascular, and cerebrovascular hospitalizations [1,2,3,30]. However, we observed non-conventional yet noteworthy results, such as the statistically insignificant association of PM_2.5_ on cardiovascular EAD outcomes, and an inverse association with cerebrovascular EAD outcomes. Also, the effect estimates of PM_2.5_ on EAD did not vary largely among age categories.

We found that the effect of ambient PM_2.5_ was significant in respiratory outcomes but not in cardiovascular outcomes. This is consistent with findings reported in previous studies [2,31,32], though one paper emphasized the effect modification by PM_2.5_ components [31]. Another paper reported that ambient PM_2.5_ increased the risk for various acute respiratory outpatient visits, including upper respiratory tract infections, acute bronchitis, community-acquired pneumonia, and acute exacerbation of bronchiectasis [32]. Since the respiratory system is among the first to be exposed and injured upon exposure to ambient PM_2.5_, the pathophysiological pathway of its effects may lead to systemic damages. Earlier studies suggested that particulate exposure could lead to cell injury due to oxidative stress [33], inflammatory responses [34], and imbalanced intracellular calcium homeostasis [35]. These pro-inflammatory conditions due to exposure to air pollutant could be underlying mechanisms for other diseases such as cardiovascular and neuropsychological outcomes.

The number of studies focusing on the association of air pollution with neuropsychological outcomes have increased in recent years, as inflammation and oxidative stress caused by air pollution are thought to cause neurotoxic effects [17,18,20]. There are several possible pathways to the brain: (1) direct transportation of pollutant via circulatory pathway to the brain, passing through the BBB; (2) systemic inflammation whereby cytokines were circulated to the brain; and (3), the nose-to-brain route that bypasses the protective BBB whereby pollutants are transported along the olfactory nerve to the brain [36], prior to the underlying toxicity mechanisms such as inflammation, microglial activation [37], oxidative stress, and neuronal death [38]. A few recent studies found that long-term exposure to ambient PM_2.5_ was associated with increased risks in Alzheimer’s disease, Parkinson’s disease [18], dementia [18,19] and memory loss [20]. There are also studies reporting the positive association between short-term exposure to PM_2.5_ and Parkinson’s disease [4,16] and headache [17]. Interestingly, Chen et al. (2015) [39] found increased clinic visits for migraine associated with ambient PM_2.5_ on warm days but not on cold days.

The statistically insignificant result exhibited by the cardiovascular outcomes in our study is similar to those reported in the previous studies carried out in Japan [10,12]. Pulmonary inflammation leads to pathways, including systemic inflammatory responses, an increase in blood coagulation factors, and an imbalanced autonomic reflex which interferes with the hearth rhythm control [15]. Ichiki et al. (2016) suggested that the possibility of the effect attenuation was due to the inclusion of a wide range of age or a lower incidence of underlying ischemic heart diseases in Japan, compared to Western countries [12]. In Sajani et al. (2014), PM_10_-related all-cause EAD was higher when compared with all-cause mortality, with cardiovascular and respiratory EAD being lower when compared to that of mortality and hospital admissions [9]. Further investigations would be necessary to determine whether the use of EAD underestimates certain types of disease or misclassifies study populations.

In contrast with previous findings regarding the effects of ambient PM_2.5_ on cerebrovascular outcomes [40,41], our study observed inverse associations, especially on average lags. There are three possibilities which could explain this situation. The first possibility is that the effect was too acute, or that the frail population was too weak, that the incidence rate was lower than the fatality rate, causing a low prevalence in the study population [42]. On the other hand, it could be due to the harvesting effect, which is also known as the displacement of a health outcome (in this study, EAD displacement). Wellenius et al. (2012) [30] reported a 12-h lag in PM_2.5_ on stroke onset, instead of a per-day lag. If stroke is triggered within 24 h, the use of a per-day lag might attenuate the effect estimates (Figure S1). The third possibility is the displacement of the frail population into other categories. In mortality studies, double-counting of cases is not a concern. Thus, in observing mortality displacement, category displacement would not be an issue. In the utilization of EAD as a health indicator, category displacement could be an issue, in addition to the over-time morbidity displacement. This is possible as cerebrovascular patients could have complications, such as recurrent stroke, seizure, pain, anxiety depression, or infection [43,44]. Assuming that long-term exposure to ambient PM_2.5_ has predisposed an individual to a morbid condition, the partial effects observed here might be due to the harvesting effect, even if it is not a total attribute. This would also explain the insignificance of observations in cardiovascular outcomes in our study. These, however, require further investigation.

The lag pattern observed in the current study is consistent with the hypothesized pattern of the harvesting effect, whereby a drop follows an initial increase. Although the drops were significant, as observed in Figure 3a and Figure S1, the pattern of a drop following the initial increase reflected the possibility that health outcomes were brought forward in time. In other words, if the initial increase was counterbalanced by the drop, there is a harvesting effect in the association being observed. Otherwise, a second increase would follow for the drop, whereby all three parts throughout the lags could provide an overview of the association—air pollution does not only harvest (displace) frail populations, but also increases the pool size of frail populations [45].

This study has several strengths. First, the multi-city analysis allowed a representative overview of the association between ambient PM_2.5_ and various health outcomes. There were significant differences of environmental variables between cities (Table S3), thus pooling the effect estimates covering the geologically distinct locations improved the representativeness of the observed health effects of PM_2.5_. Although another study focusing on cardiovascular disease in Japan included over 30 prefectures, it only included data over 9 months [12], whereas our study spanned a longer period. Evidence of adverse effects of short-term exposure to ambient PM_2.5_ on EAD for all-cause, respiratory, and neuropsychological outcomes in Japan was added to the literature. Second, we explored the 7-day lag structure, which allowed us to observe potential harvesting effects as well as average effects in different health outcomes. Third, our study indicated the usefulness of EAD as an indicator, which reflects an acute onset of medical conditions. Although EAD has not been used until recently, establishing the consistency and reliability of EAD as a health indicator is important for future studies in countries where other health data are difficult to obtain.

Nonetheless, we would like to acknowledge some limitations in our study. First, the diagnosis for each dispatch was confirmed by a medical doctor upon arrival of the dispatch. This might misclassify the exact medical condition which would be diagnosed afterwards. It is not possible for us to detect the misclassification, if there is any, as our data did not include any individual information. Second, exposure to ambient PM_2.5_ might have been misclassified as our data could not represent the actual personal exposure for each individual. Furthermore, air pollutant data was obtained from one monitoring station in each city. Third, the EAD data contained general categories of diagnoses. Although it is useful in providing a generalizable and representative observation on the study population, it could not show the effect on specific types of disease from each category of diagnoses. For example, the EAD data could provide a population within the category of respiratory outcomes, but it could not distinguish between specific types of respiratory outcomes, such as bronchitis, asthma, or chronic obstructive pulmonary disease. Finally, more recent data were not available, despite efforts trying to update it. More recent data might contribute additional information, although it may not significantly alter the slope of the risk.

## 5. Conclusions

In conclusion, short-term exposure to ambient PM_2.5_ increased the risk of EAD for all-cause, respiratory, and neuropsychological outcomes. The effects observed were generally acute, occurring within the first 2 days. Average lag effects were observed only in respiratory outcomes, extending up to lag 0–3. No significant association was observed in cardiovascular outcomes, while inverse associations were observed in cerebrovascular outcomes.

## Figures and Tables

**Table 1:** Daily average number of emergency ambulance dispatches (EAD) in each city during the study period.

Characteristics	Sapporo	Sendai	Saitama	Nagoya	Osaka	Hiroshima	Fukuoka	Kumamoto
**Study period**	1 January 2007–31 March 2010	1 January 2007–31 March 2010	1 April 2009–31 March 2011	1 January 2008–31 December 2011	1 January 2008–31 December 2011	1 April 2010–31 December 2011	1 January 2009–31 December 2011	1 April 2010–31 December 2011
Population *	1,913,545	1,045,986	1,222,434	2,263,894	2,665,314	1,173,843	1,463,743	734,474
**Sex**								
Male	53 (9)	28 (6)	46 (9)	NA	NA	NA	46 (8)	21 (5)
Female	58 (9)	27 (6)	41 (8)	NA	NA	NA	47 (8)	22 (5)
**Age**								
Children	8 (4)	4 (2)	8 (4)	9 (5)	21 (7)	6 (3)	7 (3)	3 (2)
Adult	53 (9)	24 (5)	37 (8)	66 (12)	142 (18)	31 (7)	43 (8)	18 (4)
Elderly	51 (8)	27 (6)	41 (9)	84 (15)	142 (19)	38 (7)	43 (8)	23 (5)
**Diagnosis type**								
All acute illness	111 (14)	55 (9)	86 (14)	159 (22)	305 (33)	74 (11)	93 (13)	43 (8)
Cardiovascular	14 (4)	4 (2)	4 (2)	15 (4)	31 (6)	10 (3)	9 (3)	3 (2)
Respiratory	12 (5)	5 (2)	5 (2)	18 (5)	32 (7)	10 (3)	12 (4)	3 (1)
Cerebrovascular	18 (6)	5 (2)	5 (2)	11 (4)	25 (5)	12 (4)	24 (5)	3 (2)
Neuropsychology	12 (4)	5 (2)	6 (3)	14 (4)	53 (9)	10 (3)	5 (2)	3 (2)

* Population based on Japanese census in 2010. Values are shown as the daily mean (standard deviation). Note: Data on the sex category were not available in Nagoya, Osaka, nor Hiroshima.

**Table 2:** Daily average concentration of environmental variables in each city during the study period.

Environmental Variable	Sapporo	Sendai	Saitama	Nagoya	Osaka	Hiroshima	Fukuoka	Kumamoto
PM_2.5_ (µg/m^3^)	11.27 (5.71)	12.41 (6.59)	17.86 (11.76)	16.00 (8.42)	18.58 (9.90)	20.84 (12.32)	18.14 (10.31)	18.73 (12.96)
SO_2_ (ppb)	2.27 (1.57)	0.59 (0.58)	1.33 (0.62)	1.83 (1.18)	5.22 (2.97)	1.24 (0.86)	1.77 (1.19)	3.41 (1.56)
NO_2_ (ppb)	15.53 (9.78)	13.83 (6.09)	18.78 (7.58)	20.83 (7.72)	20.23 (9.09)	13.82 (6.44)	13.46 (7.35)	9.77 (4.92)
O_x_ (ppb)	27.58 (11.15)	29.17 (11.62)	28.47 (13.34)	24.70 (12.86)	29.00 (13.21)	27.60 (12.98)	29.45 (13.44)	26.37 (12.15)
Temperature (°C)	8.64 (9.31)	12.19 (8.00)	15.52 (8.62)	16.43 (8.56)	17.08 (8.35)	17.69 (8.57)	17.35 (8.05)	18.34 (8.31)
Relative humidity (%)	67.92 (10.23)	71.48 (13.00)	65.06 (13.84)	63.50 (12.35)	62.76 (10.98)	64.53 (10.33)	65.58 (11.96)	69.33 (10.84)

Values are shown as daily mean (standard deviation).

**Table 3:** Pooled effect of ambient PM_2.5_ on cause-specific EAD outcomes.

Lag Structure	Cardiovascular	Respiratory	Cerebrovascular	Neuropsychology
Lag 0 ^a^	0.36 (−1.30, 2.05)	1.88 (1.00, 2.76) *	0.24 (−0.62, 1.11)	1.48 (0.69, 2.28) *
**Average lags**				
Lag 0–1	−0.10 (−0.92, 0.73)	2.47 (1.69, 3.26) *	−1.76 (−2.80, −0.72) *	0.59 (−1.09, 2.30)
Lag 0–3	−0.19 (−1.47, 1.11)	2.79 (1.31, 4.29) *	−1.27 (−2.22, −0.32) *	1.03 (−1.71, 3.84)
Lag 0–5	−0.20 (−1.37, 0.98)	1.86 (0.23, 3.51) *	−1.13 (−2.23, −0.02) *	0.06 (−2.50, 2.68)
Lag 0–7	−0.60 (−1.90, 0.72)	1.53 (−0.07, 3.16)	−1.05 (−2.29, 0.19)	−0.30 (−2.71, 2.17)

Values were shown as percent change (95% CI). ^a^ Lag 0 of unconstrained, distributed lag model. * Statistical significance at *p* < 0.05.

**Table 4:** Pooled effect of ambient PM_2.5_ on all-cause EAD by age category.

Lag Structure	Children (Age below 18 Years)	Adult (Age 18 to 64 Years)	Elderly (Age 65 Years and above)
Lag 0 ^a^	1.24 (0.21, 2.27) *	1.29 (0.87, 1.71) *	1.19 (0.75, 1.62) *
**Average lags**			
Lag 0–1	0.89 (−0.09, 1.89)	0.78 (0.25, 1.32) *	0.65 (0.20, 1.11) *
Lag 0–3	1.09 (−0.14, 2.32)	0.55 (−0.37, 1.48)	0.61 (0.16, 1.07) *
Lag 0–5	0.45 (−0.96, 1.88)	0.16 (−0.99, 1.33)	0.34 (−0.36, 1.05)
Lag 0–7	0.03 (−1.53, 1.61)	0.26 (−0.93, 1.47)	0.05 (−0.73, 0.84)

Values are shown as percent change (95% CI). ^a^ Lag 0 of unconstrained, distributed lag model. * Statistical significance at *p* < 0.05.

## Supplementary Materials

The following are available online at http://www.mdpi.com/1660-4601/15/2/307/s1, Table S1: Sensitivity analysis using different df on calendar date, Table S2: Sensitivity analysis using different df on temperature, Table S3: *p*-values of Tukey’s test for environmental variables between cities, Table S4: Amount of heterogeneity (*I*^2^) (%) for each diagnosis at different lags. Figure S1: Percent change of EAD associated with 10 µg/m^3^ increase in PM_2.5_ in unconstrained, distributed lag model.

## References

[1] Bell M.L., Ebisu K., Peng R.D., Walker J., Samet J.M., Zeger S.L., Dominici F. (2008). Seasonal and Regional Short-term Effects of Fine Particles on Hospital Admissions in 202 US Counties, 1999–2005. Am. J. Epidemiol..

[2] Dominici F., Peng R.D., Bell M.L., Pham L., McDermott A., Zeger S.L., Samet J.M. (2006). Fine particulate air pollution and hospital admission for cardiovascular and respiratory diseases. JAMA.

[3] Stafoggia M., Samoli E., Alessandrini E., Cadum E., Ostro B., Berti G., Faustini A., Jacquemin B., Linares C., Pascal M. (2013). Short-term associations between fine and coarse particulate matter and hospitalizations in southern Europe: Results from the MED-PARTICLES project. Environ. Health Perspect..

[4] Zanobetti A., Dominici F., Wang Y., Schwartz J.D. (2014). A national case-crossover analysis of the short-term effect of PM_2.5_ on hospitalizations and mortality in subjects with diabetes and neurological disorders. Environ. Health.

[5] Bravo M.A., Ebisu K., Dominici F., Wang Y., Peng R.D., Bell M.L. (2017). Airborne Fine Particles and Risk of Hospital Admissions for Understudied Populations: Effects by Urbanicity and Short-Term Cumulative Exposures. Environ. Health Perspect..

[6] Metzger K.B., Tolbert P.E., Klein M., Peel J.L., Flanders W.D., Todd K., Mulholland J.A., Ryan P.B., Frumkin H. (2004). Ambient air pollution and cardiovascular emergency department visits. Epidemiology.

[7] Atkinson R., Kang S., Anderson H., Mills I., Walton H. (2014). Epidemiological time series studies of PM_2.5_ and daily mortality and hospital admissions: A systematic review and meta-analysis. Thorax.

[8] Wong C.-M., Vichit-Vadakan N., Kan H., Qian Z., Teams P.P. (2008). Public health and air pollution in Asia (PAPA): A multicity study of short-term effects of air pollution on mortality. Environ. Health Perspect..

[9] Sajani S.Z., Alessandrini E., Marchesi S., Lauriola P. (2014). Are day-to-day variations of airborne particles associated with emergency ambulance dispatches?. Int. J. Occup. Environ. Health.

[10] Michikawa T., Ueda K., Takeuchi A., Kinoshita M., Hayashi H., Ichinose T., Nitta H. (2015). Impact of short-term exposure to fine particulate matter on emergency ambulance dispatches in Japan. J. Epidemiol. Community Health.

[11] Tasmin S., Ueda K., Stickley A., Yasumoto S., Phung V.L.H., Oishi M., Yasukouchi S., Uehara Y., Michikawa T., Nitta H. (2016). Short-term exposure to ambient particulate matter and emergency ambulance dispatch for acute illness in Japan. Sci. Total Environ..

[12] Ichiki T., Onozuka D., Kamouchi M., Hagihara A. (2016). An association between fine particulate matter (PM_2.5_) levels and emergency ambulance dispatches for cardiovascular diseases in Japan. Int. Arch. Occup. Environ. Health.

[13] Liu R., Zeng J., Jiang X., Chen J., Gao X., Zhang L., Li T. (2017). The relationship between airborne fine particulate matter and emergency ambulance dispatches in a southwestern city in Chengdu, China. Environ. Pollut..

[14] Salimi F., Henderson S.B., Morgan G.G., Jalaludin B., Johnston F.H. (2017). Ambient particulate matter, landscape fire smoke, and emergency ambulance dispatches in Sydney, Australia. Environ. Int..

[15] Brook R.D., Rajagopalan S., Pope C.A., Brook J.R., Bhatnagar A., Diez-Roux A.V., Holguin F., Hong Y., Luepker R.V., Mittleman M.A. (2010). Particulate Matter Air Pollution and Cardiovascular Disease. An Update to the Scientific Statement from the American Heart Association. Circulation.

[16] Lee H., Myung W., Kim D.K., Kim S.E., Kim C.T., Kim H. (2017). Short-term air pollution exposure aggravates Parkinson’s disease in a population-based cohort. Sci. Rep..

[17] Szyszkowicz M. (2008). Ambient air pollution and daily emergency department visits for headache in Ottawa, Canada. Headache.

[18] Kioumourtzoglou M., Schwartz J.D., Weisskopf M.G., Melly S.J., Wang Y., Dominici F., Zanobetti A. (2016). Long-term PM_2.5_ exposure and neurological hospital admissions in the northeastern United States. Environ. Health Perspect..

[19] Chen H., Kwong J.C., Copes R., Hystad P., van Donkelaar A., Tu K., Brook J.R., Goldberg M.S., Martin R.V., Murray B.J. (2017). Exposure to ambient air pollution and the incidence of dementia: A population-based cohort study. Environ. Int..

[20] Chen J.-C., Wang X., Wellenius G.A., Serre M.L., Driscoll I., Casanova R., McArdle J.J., Manson J.E., Chui H.C., Espeland M.A. (2015). Ambient Air Pollution and Neurotoxicity on Brain Structure: Evidence from Women’s Health Initiative Memory Study. Ann. Neurol..

[21] Murray C.J.L., Lopez A.D. (1996). Summary. The Global Burden of Disease: A Comprehensive Assessment of Mortality and Disability from Diseases, Injuries and Risk Factors in 1990 and Projected to 2020.

[22] Block M.L., Calderón-Garcidueñas L. (2009). Air pollution: Mechanisms of neuroinflammation and CNS disease. Trends Neurosci..

[23] Tanigawa K., Tanaka K. (2006). Emergency medical service systems in Japan: Past, present, and future. Resuscitation.

[24] Ng C.F.S., Ueda K., Takeuchi A., Nitta H., Konishi S., Bagrowicz R., Watanabe C., Takami A. (2014). Sociogeographic variation in the effects of heat and cold on daily mortality in Japan. J. Epidemiol..

[25] Dominici F., Samet J.M., Zeger S.L. (2000). Combining evidence on air pollution and daily mortality from the 20 largest US cities: A hierarchical modelling strategy. J. R. Stat. Soc. Ser. A Stat. Soc..

[26] Chen R., Chu C., Tan J., Cao J., Song W., Xu X., Jiang C., Ma W., Yang C., Chen B. (2010). Ambient air pollution and hospital admission in Shanghai, China. J. Hazard. Mater..

[27] Higgins J.P.T., Thompson S.G. (2002). Quantifying heterogeneity in a meta-analysis. Stat. Med..

[28] R Core Team (2014). R: A Language and Environment for Statistical Computing.

[29] Viechtbauer W. (2010). Conducting Meta-Analyses in R with the metafor Package. J. Stat. Softw..

[30] Wellenius G.A., Burger M.R., Coull B.A., Schwartz J., Suh H.H., Koutrakis P., Schlaug G., Gold D.R., Mittleman M.A. (2012). Ambient air pollution and the risk of acute ischemic stroke. Arch. Intern. Med..

[31] Zanobetti A., Franklin M., Koutrakis P., Schwartz J. (2009). Fine particulate air pollution and its components in association with cause-specific emergency admissions. Environ. Health.

[32] Li R., Jiang N., Liu Q., Huang J., Guo X., Liu F. (2017). Impact of Air Pollutants on Outpatient Visits for Acute Respiratory Outcomes. Int. J. Environ. Res. Public Health.

[33] Donaldson K., Beswick P.H., Gilmour P.S. (1996). Free radical activity associated with the surface of particles: A unifying factor in determining biological activity?. Toxicol. Lett..

[34] Seriani R., de Souza C.E.C., Krempel P.G., Frias D.P., Matsuda M., Correia A.T., Ferreira M.Z.J., Alencar A.M., Negri E.M., Saldiva P.H.N. (2016). Human bronchial epithelial cells exposed in vitro to diesel exhaust particles exhibit alterations in cell rheology and cytotoxicity associated with decrease in antioxidant defenses and imbalance in pro- and anti-apoptotic gene expression. Environ. Sci. Pollut. Res..

[35] Brown D.M., Donaldson K., Borm P.J., Schins R.P., Dehnhardt M., Gilmour P., Jimenez L.A., Stone V. (2004). Calcium and ROS-mediated activation of transcription factors and TNF-alpha cytokine gene expression in macrophages exposed to ultrafine particles. Am. J. Physiol. Lung Cell. Mol. Physiol..

[36] Lucchini R., Dorman D., Elder A., Veronesi B. (2012). Neurological impacts from inhalation of pollutants and the nose-brain connection. Neurotoxicology.

[37] Block M.L., Zecca L., Hong J.-S. (2007). Microglia-mediated neurotoxicity: Uncovering the molecular mechanisms. Neuroscience.

[38] Heneka M.T., Kummer M.P., Latz E. (2014). Innate immune activation in neurodegenerative disease. Nat. Rev. Immunol..

[39] Chen C., Tsai S., Yang C. (2015). Association between fine particulate air pollution and daily clinic visits for migraine in a subtropical city: Taipei, Taiwan. Int. J. Environ. Res. Public Health.

[40] Ljungman P.L., Mittleman M.A. (2014). Ambient air pollution and stroke. Stroke.

[41] Sade M.Y., Novack V., Ifergane G., Horev A., Kloog I. (2015). Air pollution and ischemic stroke among young adults. Stroke.

[42] Wilkinson P., Milner J., Armstrong B. Other cardiovascular effects of ambient air pollution [abstract]. Proceedings of the 29th Annual Science Conference International Society of Environmental Epidemiology.

[43] Davenport R.J., Dennis M.S., Wellwood I., Warlow C.P. (1996). Complications after acute stroke. Stroke.

[44] Langhorne P., Stott D.J., Robertson L., MacDonald J., Jones L., McAlpine C., Dick F., Taylor G.S., Murray G. (2000). Medical complications after stroke. A multicenter study. Stroke.

[45] Zanobetti A., Wand M.P., Schwartz J., Ryan L.M. (2000). Generalized additive distributed lag models: Quantifying mortality displacement. Biostatistics.

