# Supplementary material for: Acute Effects of Ambient PM2.5 on All-Cause and Cause-Specific Emergency Ambulance Dispatches in Japan
Source: Int J Environ Res Public Health. 2018 Feb 9;15(2):307. doi: 10.3390/ijerph15020307 (PMC5858376; doi:10.3390/ijerph15020307)
Supplement: Supplementary file 1 [file ijerph-15-00307-s001.pdf]

Table S1. Sensitivity analysis using different df on calendar date.

df	Percent Change (%)	95% CI
3	1.10	(0.80, 1.39)
4	1.14	(0.80, 1.47)
5	1.16	(0.88, 1.43)
6	1.18	(0.90, 1.45)
**7**	**1.20**	**(0.86, 1.53)**
8	1.25	(0.97, 1.53)
9	1.17	(0.81, 1.52)
10	1.17	(0.83, 1.50)
11	1.21	(0.89, 1.53)
12	1.23	(0.93, 1.53)
13	1.23	(0.91, 1.55)

Note: Bolded values are from current model.

Table S2. Sensitivity analysis using different df on temperature.

df	Percent Change (%)	95% CI
**3**	**1.20**	**(0.86, 1.53)**
4	1.24	(0.90, 1.58))
5	1.19	(0.85, 1.52)
6	1.20	(0.88, 1.51)
7	1.20	(0.86, 1.53)
8	1.20	(0.88, 1.51)

Note: Bolded values were from current model.

Table S3. *p*-values of Tukey's test for environmental variables between cities.

City	Environmental Variables	Sendai	Saitama	Nagoya	Osaka	Hiroshima	Fukuoka	Kumamoto
Sapporo	$PM_{2.5}$	0.0640	**<0.0001**	**<0.0001**	**<0.0001**	**<0.0001**	**<0.0001**	**<0.0001**
	SO_2	**<0.0001**	**<0.0001**	**<0.0001**	**<0.0001**	**<0.0001**	**<0.0001**	**<0.0001**
	NO_2	**<0.0001**	**<0.0001**	**<0.0001**	**<0.0001**	**0.0002**	**<0.0001**	**<0.0001**
	O_x	**0.0484**	0.8059	**<0.0001**	0.0768	>0.9999	**0.0097**	0.5139
	Temperature	**<0.0001**	**<0.0001**	**<0.0001**	**<0.0001**	**<0.0001**	**<0.0001**	**<0.0001**
	RH	**<0.0001**	**<0.0001**	**<0.0001**	**<0.0001**	**<0.0001**	**<0.0001**	0.2207
Sendai	$PM_{2.5}$	-	**<0.0001**	**<0.0001**	**<0.0001**	**<0.0001**	**<0.0001**	**<0.0001**
	SO_2	-	**<0.0001**	**<0.0001**	**<0.0001**	**<0.0001**	**<0.0001**	**<0.0001**
	NO_2	-	**<0.0001**	**<0.0001**	**<0.0001**	>0.9999	0.9452	**<0.0001**
	O_x	-	0.9421	**<0.0001**	>0.9999	0.2149	0.9994	**0.0002**
	Temperature	-	**<0.0001**	**<0.0001**	**<0.0001**	**<0.0001**	**<0.0001**	**<0.0001**
	RH	-	**<0.0001**	**<0.0001**	**<0.0001**	**<0.0001**	**<0.0001**	**0.0048**
Saitama	$PM_{2.5}$		-	**0.0005**	0.7192	**<0.0001**	0.9989	0.7309
	SO_2		-	**<0.0001**	**<0.0001**	0.9508	**<0.0001**	**<0.0001**
	NO_2		-	**<0.0001**	**0.0012**	**<0.0001**	**<0.0001**	**<0.0001**
	O_x		-	**<0.0001**	0.9837	0.9156	0.7340	**0.0435**
	Temperature		-	0.2647	**0.0012**	**0.0001**	**0.0002**	**<0.0001**
	RH		-	0.0675	**0.0005**	0.9920	0.9884	**<0.0001**
Nagoya	$PM_{2.5}$			-	**<0.0001**	**<0.0001**	**<0.0001**	**<0.0001**
	SO_2			-	**<0.0001**	**<0.0001**	0.9762	**<0.0001**

	NO_2	-	0.4150	**<0.0001**	**<0.0001**	**<0.0001**
	O_x	-	**<0.0001**	**0.0001**	**<0.0001**	0.0957
	Temperature	-	0.4120	**0.0337**	0.1279	**0.0001**
	RH	-	0.7013	0.5785	**0.0003**	**<0.0001**
Osaka	$PM_{2.5}$		-	**<0.0001**	0.9390	>0.9999
	SO_2		-	**<0.0001**	**<0.0001**	**<0.0001**
	NO_2		-	**<0.0001**	**<0.0001**	**<0.0001**
	O_x		-	0.3058	0.9865	**0.0003**
	Temperature		-	0.7967	0.9959	**0.0388**
	RH		-	**0.0329**	**<0.0001**	**<0.0001**
Hiroshima	$PM_{2.5}$			-	**<0.0001**	**0.0037**
	SO_2			-	**<0.0001**	**<0.0001**
	NO_2			-	0.9831	**<0.0001**
	O_x			-	0.0779	0.6892
	Temperature			-	0.9896	0.8780
	RH			-	0.6664	**<0.0001**
Fukuoka	$PM_{2.5}$				-	0.9264
	SO_2				-	**<0.0001**
	NO_2				-	**<0.0001**
	O_x				-	**<0.0001**
	Temperature				-	0.2519
	RH				-	**<0.0001**

Note: Significant differences at $p<0.005$ are bolded. RH denotes relative humidity.

Table S4. Amount of heterogeneity (I^2) (%) for each diagnosis at different lags

Lag structure	All cause	Cardiovascular	Respiratory	Cerebrovascular	Neuropsychology
**Unconstrained distributed lag**					
0	10.65	59.33*	2.25	1.13	1.40
1	2.24	0.00	0.00	0.16	0.00
2	48.96	0.20	0.00	24.82	20.37
3	0.00	0.00	0.83	0.00	19.66
4	3.38	0.00	0.70	45.65	0.32
5	0.00	0.00	17.42	1.74	13.63
6	0.01	18.84	4.17	0.00	35.96
7	2.03	0.00	44.44	0.00	0.00
**Average lag**					
0-1	51.96	2.37	1.37	34.36	0.00
0-3	74.91*	21.61	38.10	0.00	35.76
0-5	75.14*	0.00	32.20	0.00	56.66
0-7	68.94*	0.00	20.24*	0.00	46.00

* Statistical significance at $p<0.05$.

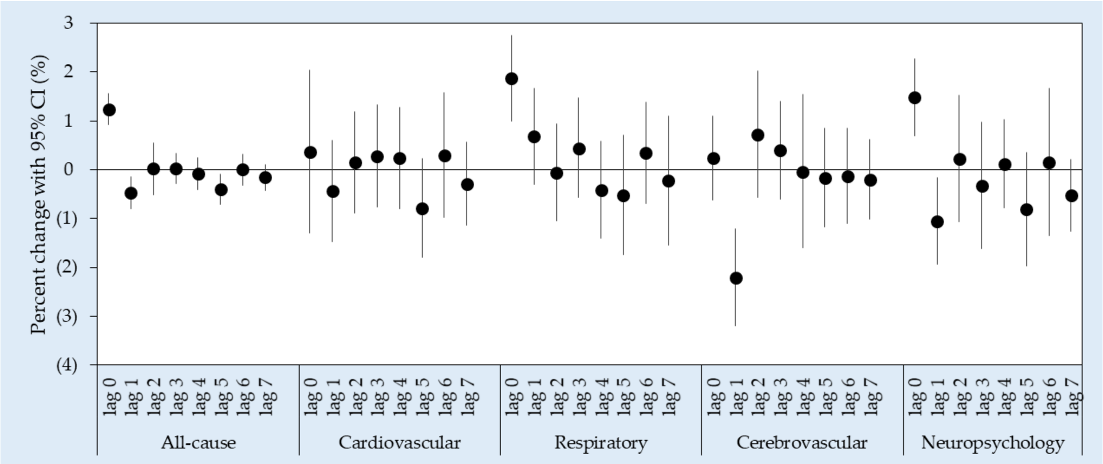

Figure S1. Percent change of EAD associated with 10 μg/m³ increase in $PM_{2.5}$ in unconstrained, distributed lag model.
